# Books and Magazines

**Published:** 1909-10

**Authors:** 


### Books and Magazines.

THE ANNALS OF SURGERY FOR JULY, 366 pages, $5.00 a year, 50 cents a copy. J. B. Lippincott Co., Philadelphia.

This large issue is the American Surgical Association number of the Philadelphia meeting, June 3-5. There are thirty-six papers presented with brief discussions. Several foreign writers read papers. Professor Friedrich, of Marburg, on the "Operative Treatment of Pulmonary Tuberculosis," and Mr. W. Arbuthnot Lane, of London, on "Chronic Intestinal Stasis." Ransohoff, of Cincinnati, read a paper on "Gunshot Wounds of the Brain." Indiana was not represented in the restricted list of papers which no doubt add worthy material to American surgery.

Parenthood and Race Culture. An Outline of Eugenics. By Caleb Williams Saleeby, M. D., Ch. B., F. Z. R. (Edin.); Fellow of the Obstetrical Society of Edinburgh; Member of Council of the Eugenics Education Society, the Sociological Society, the National League for Physical Education and Improvement; Member of the Royal Institution, the Society for the Study of Inebriety, etc.

This book constitutes the first attempt to define, as a whole, the general principles of race culture or eugenics. The author assumes that there is no wealth but life, that the culture of the racial life is the vital industry of any people, that conditions of parenthood, and especially as regards its quality rather than its quantity, are the dominant factors that determine the destiny of nations.

Defining the limits of education, and recognizing the importance of heredity, he seeks to show how eugenics may be practiced even in the present state of social sentiment, and how marriage may serve, with enhanced value, to this end. The principles of what the author calls negative eugenics—the discouragement of parenthood on the part of the insane, chronic enebriates and feeble-minded—are carefully discussed.

---

Report of the Commissioner of Health of Pennsylvania. 1908. Harrisburg, Pa.

We acknowledge receipt of a copy of this voluminous report. It should be in the library of every State Health Department and public library.

RITCHIE'S HUMAN PHYSIOLOGY. An Elementary Text-Book of Anatomy, Physiology and Hygiene. By John W. Ritchie, Professor of Biology, College of William and Mary, Virginia. Illustrated by Mary H. Wellman. World Book Co., Yonkers-on-Hudson, New York. 1909. Cloth. Mailing price, 96 cents.

This is one of a series of school books published by this company. Its value can scarcely be overestimated, for it meets the requirements of an elementary, though full, text-book on the subject, adapted to the understanding of girls and boys. It were supererogation to even allude this late day to the importance of instructing children in physiology and hygiene. The TEXAS MEDICAL JOURNAL approves and recommends this book to all who are interested in the education of the young.

